# Extensive Bone Lengthening for a Patient with Linear Morphea

**DOI:** 10.1155/2018/4535804

**Published:** 2018-08-19

**Authors:** Kenichi Mishima, Hiroshi Kitoh, Masaki Matsushita, Tadashi Nagata, Yasunari Kamiya, Naoki Ishiguro

**Affiliations:** Department of Orthopaedic Surgery, Nagoya University Graduate School of Medicine, 65 Tsurumai, Showa-ku, Nagoya, Aichi 466-8550, Japan

## Abstract

Localized scleroderma, also known as morphea, is a rare condition characterized by progressive sclerosis of the skin and associated atrophy of the underlying tissues. The linear type of localized scleroderma is the most frequent form in childhood, usually affecting unilateral extremities. Fibrosclerosis of the fasciae and muscles can spread across joints and impair the range of motion of the joint. Dysplastic and/or atrophic bones of the affected lower extremity can lead to clinically significant leg length discrepancy (LLD). Limb reconstruction surgery has rarely been indicated for LLD in patients with linear morphea. We report on a case of extensive bone lengthening for appreciable LLD in a pediatric patient with linear morphea. A Japanese girl with linear morphea underwent staged simultaneous lengthening of the femur and tibia twice at seven and eleven years of age using a unilateral external fixator. A healing index exceeded 100 days/cm except for the first femoral lengthening that was complicated by regenerate fracture. At the final follow-up, LLD of 38 mm remained, but she could walk independently without a brace or a crutch. Due to soft tissue tightness and poor regenerative ability in the affected limb, cautions should be taken to prevent regenerate fracture and/or malalignment of the limb.

## 1. Introduction

Localized scleroderma (LS), also known as morphea, is a rare condition characterized by progressive sclerosis of the skin and associated atrophy of the underlying tissues [1]. LS is classified into five types, including plaque type, generalized type, bullous type, linear type, and deep type [2]. The linear type is the most frequent form of LS in childhood, usually affecting unilateral extremities [3]. Fibrosclerosis of the fasciae and muscles can spread across joints and impair the range of motion of the joint. Dysplastic and/or atrophic bones of the affected lower extremity can lead to clinically significant leg length discrepancy (LLD) [4]. Severe joint contracture and LLD inevitably cause significant physical disability in patients with the linear type of LS, linear morphea (LM), for whom bone shortening or amputation has been indicated, whereas extensive bone lengthening has rarely performed. To our knowledge, there is only one case report describing limb reconstruction surgery in a patient with LM [5]. We report on a juvenile LM case who underwent extensive bone lengthening to correct substantial LLD.

## 2. Case Presentation

**Figure 1:**
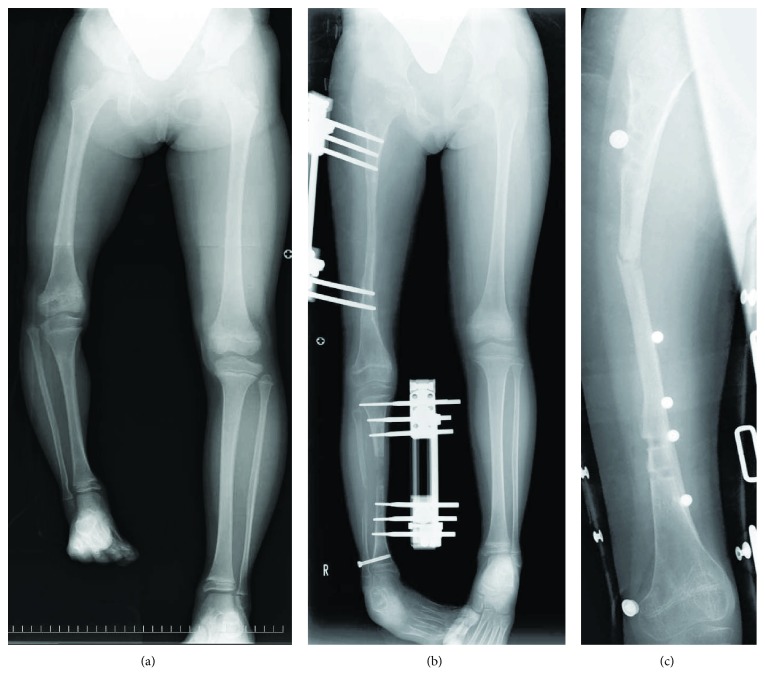
Radiographs of the lower extremities at the time of the first lengthening. (a) An anteroposterior standing radiograph of the lower extremities at seven years of age showing LLD of nearly 10 cm with the right foot placed on a block. (b) An anteroposterior supine radiograph of the lower extremities at the end of the lengthening period of the first lengthening demonstrating 83 mm and 37 mm of lengthening in the femur and tibia, respectively. (c) An anteroposterior radiograph of the right femur showing regenerate fracture at the site of the lengthened callus.

A four-year-old Japanese girl with no remarkable medical history was referred to our orthopedic clinic for treatment of 2 cm of LLD. She had a two-year history of progressive LM in a wide range of the posteromedial aspect of the right thigh and the medial aspect of the right lower leg. At the first presentation, skin lesions exhibited hyperpigmentation, induration, and xerosis. The range of motion of the right knee was full extension to 80° of flexion. Radiographs of the right lower extremity revealed dysplastic/atrophic femur and tibia. LLD increased with time and reached nearly 10 cm at seven years of age (Figure 1(a)). As she and her parents refused to undergo epiphysiodesis of the unaffected side of the lower extremity, we performed simultaneous lengthening of the right femur and tibia using a unilateral external fixator (EBI/Zimmer Biomet Carbon Rail Deformity System; Warsaw, Indiana, USA). She had taken low-dose prednisolone every day or every second day prior to the first lengthening procedure. The dosage regimen had been dependent on the disease activity based on clinical and thermographic assessment. Tibial osteotomy was performed with the Gigli saw, whereas femoral osteotomy was done with a multiple drilling technique. No postoperative immobilization was used, and full-weight bearing was encouraged from the second postoperative day. After 14 days of the waiting period, distraction of the femur and tibia was commenced at a rate of 1 mm and 0.5 mm per day, respectively. Femur was lengthened at the same rate throughout the distraction period, whereas the distraction speed of the tibia was gradually decreased after the lengthening callus showed thin and sparse on radiographs. Distraction of the tibia was occasionally interrupted until the callus width and continuity were reestablished. As a result, the lengthening period/amount of lengthening of the femur and tibia were 90 days/83 mm and 163 days/37 mm, respectively, and an overall leg length was 7 mm longer in the affected limb at the end of the lengthening period (Figure 1(b)). During the neutralizing period, an accordion technique and daily low-intensity pulsed ultrasound (LIPUS) exposure were applied to the tibia to stimulate callus maturation. She received LIPUS treatment using a sonic accelerated fracture healing system (SAFHS; Teijin Pharma Ltd., Tokyo, Japan) once a day for 20 minutes without interruption. After 84 days and 194 days of the neutralizing period in the femur and tibia, respectively, the device was loosened to allow dynamization of the lengthened callus so that it could fully mature. The dynamization period reached 49 days in the femur and 58 days in the tibia to obtain matured callus exhibiting fusiform/cylindrical shape and similar density to that of the adjacent cortical bone on radiographs. Before pin removal, we dislodged the fixator frame with the fixation pins leaving in situ for a while to monitor the development of regenerate bone fracture or bending. The monitoring period was 47 days for the tibia and only one day for the femur, because the femoral pins had already been loosened. A healing index (HI) was 29 days/cm and 129 days/cm in the femur and tibia, respectively. Regenerate fracture of the femur, however, occurred due to minor trauma three days after the pin removal (Figure 1(c)). Since parental consent for open reduction and internal fixation was not obtained, she was treated conservatively with skin traction, resulting in malunion associated with a marked anterolateral bowing.

**Figure 2:**
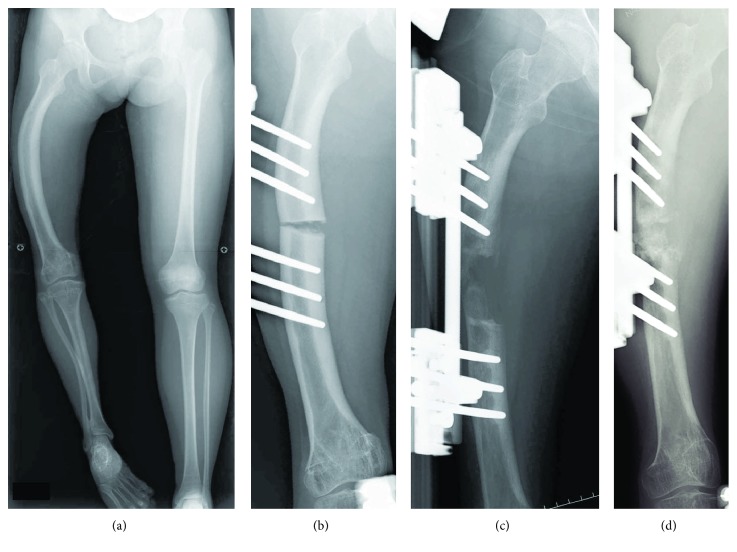
Radiographs of the lower extremities at the time of the second lengthening. (a) An anteroposterior supine radiograph of the lower extremities at eleven years of age before the second lengthening revealing LLD of 11 cm. (b) A radiograph of the right femur just after the femoral osteotomy demonstrating acute correction of an anterolateral bowing deformity. (c, d) Radiographs of the right femur before (c) and after (d) the chipping surgery.

**Figure 3:**
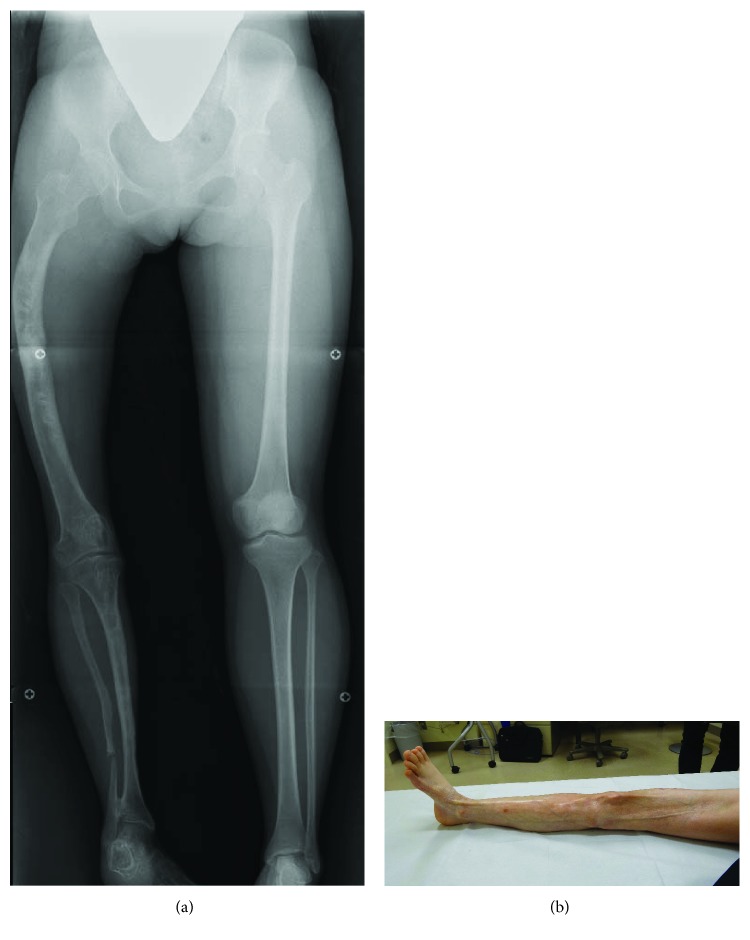
Current radiograph and view of the lower extremities. (a) An anteroposterior supine radiograph of the lower extremities at 17 years of age showing an anterolateral bowing of the right femur and a medial bowing of the right tibia. (b) A postoperative view of the right lower extremity showing atrophic skin surfaces on the posteromedial aspect of the thigh and the medial aspect of the lower leg, where brown pigmented cutaneous lesions were observed at the first presentation.

After the first lengthening procedure, LLD gradually increased again and reached 11 cm at eleven years of age (Figure 2(a)), when the flexion angle of the right knee decreased to 30 degrees. The second simultaneous lengthening of the femur and tibia was performed through percutaneous osteotomy using a multiple drilling technique. In the femur, acute correction of the bowing was done at the osteotomy site with the use of a fixator. The angulation was corrected up to 25 degrees using a proximal rotational clamp, followed by mechanical realignment of the bone axis using a distal translational clamp. After correction of the angular deformity, the osteotomy site was compressed (Figure 2(b)). Distraction by 1 mm and 0.5 mm per day was initiated at 14 days postoperatively in the femur and tibia, respectively. During the lengthening period, the rate of distraction was adjusted appropriately in order not to deteriorate the continuity of the callus on radiographs. Since the callus was poorly consolidated in the femur (Figure 2(c)), a modified “chipping and lengthening technique” was performed to enhance bone regeneration at nine months postoperatively (Figure 2(d)) [6]. Briefly, both ends of the osteotomy site and the callus were drilled with a 3.0 mm Kirschner wire in advance and then broken into smaller pieces with an osteotome. Subsequently, the comminuted bones were compressed until a radiolucent area was no longer recognized. Hard callus that obliterated the medullary cavity at the ends of the osteotomy site was removed with a sharp spoon. Two weeks after the chipping surgery, the distraction was resumed at a rate of 0.5 mm per day. The lengthening period/amount of the femur and tibia were 435 days/55 mm and 209 days/29 mm, respectively, and an overall leg length was 31 mm shorter in the affected limb at the end of the lengthening period. Symptomatic pin tract infection occasionally occurred during the treatment period and was resolved with oral antibiotics without any sequelae. The HI of the femur and tibia was 182 days/cm and 222 days/cm, respectively. Currently, two or three years have passed since the final removal of the femoral or tibial pins, respectively, and 38 mm of LLD is left with acceptable lower limb alignment (Figure 3). The range of motion of the right knee is 20° of flexion and 0° of extension, but she can walk independently without a brace or a crutch. She and her parents are satisfied with the outcome despite the long treatment period.

## 3. Discussion

Unlike the nonlinear type of LS, LM can cause significant LLD due to its unilateral involvement and early onset during childhood [3]. The bone lengthening procedure has not been indicated for LM cases, partly because it was thought to increase pathological sclerosis and contracture of the affected soft tissues [4]. Besides, the affected limb is thought to possess less bone-regenerating ability since the adipose tissue, a potent source of stem cells that can differentiate into bone-forming osteoblastic cells [7], has been shown to be replaced by poorly vasculated fibrous tissues in advanced LS [3]. Growth factors and nutrient supply necessary for osteoblastic differentiation obviously require abundant vascularization [8]. Therefore, LM-associated LLD has been managed primarily by epiphysiodesis and/or bone shortening, and, in some severe cases, by limb amputation [4]. In this case, however, the patient and her parents eagerly desired limb reconstruction surgery, although we expected that soft tissue tightness and poor regenerative ability in the affected limb would hamper successful extensive bone lengthening.

Juvenile LS patients have shown higher levels of IFN-*γ* and GM-CSF and lower levels of TNF-*α* [9]. These cytokines directly or indirectly regulate osteoclastogenesis, with IFN-*γ* and GM-CSF suppressing [10, 11] and TNF-*α* activating osteoclast differentiation [12]. Osteoclast-mediated resorption of calcified tissues is indispensable for proper bone remodeling [8]. The predominance of suppressed osteoclastogenesis may thus lead to the delay in callus maturation, which is presumably responsible for regenerate fracture as occurred in the first femoral lengthening. Tibial lengthening and the second femoral lengthening, on the other hand, necessitated an extremely long treatment period but were not associated with regenerate fractures. We thus consider that acceptable bone lengthening in LM patients may require a slow rate of distraction to preserve the integrity of the lengthened callus and quite a long neutralizing and dynamization period to ensure the consolidation and maturation of the callus.

The regenerate fracture malunited, and the extension contracture of the knee deteriorated after the first femoral lengthening. A review of MRI findings of LS has shown that fascial thickening and enhancement were notable in addition to subcutaneous septal thickening [13]. In our case, contractured fasciae at the posteromedial side of the thigh could distort the fractured femur as if a strained string had bent a bow. Open reduction and osteosynthesis should be done for regenerate fractures to prevent bowing deformity and keep bone length in this specific disease.

Limb reconstruction by extensive bone lengthening can be achieved even in severe LM patients, although it may need an extremely long period of an external fixation. We thus recommend the combination of bone lengthening of the affected side and epiphysiodesis of the healthy side to minimize LLD expansion and the amount of lengthening required. In addition, we propose the conversion of external fixation to rigid internal fixation after the lengthening period not only to protect against regenerate fractures but also to shorten the period of external fixation. In severe LM cases, staged lengthening is indispensable to correct a large amount of LLD. To this end, conversion to plate fixation rather than intramedullary nailing may be preferable, because the endosteum, which is important for bone regeneration, should be preserved for a subsequent lengthening procedure [14].

We report on a rare case of extensive bone lengthening for LLD in a patient with linear morphea. Satisfactory correction of LLD can be attained even in severe cases of linear morphea by extensive bone lengthening alone despite an extremely long treatment period. Due to soft tissue tightness and poor regenerative ability in the affected limb, cautions should be taken to prevent regenerate fracture and/or malalignment of the limb.
